# Good response to pulmonary arterial hypertension-targeted therapy in 2 pulmonary veno-occlusive disease patients: A case report

**DOI:** 10.1097/MD.0000000000027334

**Published:** 2021-10-15

**Authors:** Li Zhang, Yao Wang, Ruifeng Zhang

**Affiliations:** a Department of Respiratory medicine, Zhongda Hospital of Southeast University, Nanjing, China; b Department of Endocrinology, Zhongda Hospital of Southeast University, Nanjing, China.

**Keywords:** EIF2AK4, pulmonary arterial hypertension, pulmonary veno-occlusive disease

## Abstract

**Rationale:**

Pulmonary veno-occlusive disease (PVOD) is a kind of rare and fatal pulmonary arterial hypertension (PAH). Different from other subtypes of PAH, PVOD patients have a very poor prognosis because of the progressive nature of pulmonary vascular involvement and fatal pulmonary edema induced by PAH-targeted drugs. Lung transplantation is the only choice for these patients.

**Patient concerns:**

We reported 2 cases of PVOD which was misdiagnosed as idiopathic pulmonary arterial hypertension initially due to the lack of typical findings of PVOD. Right heart catheterization was done. The results showed severe PAH with mean pulmonary artery pressure at 76 mmHg and 68 mmHg.

**Diagnosis:**

The diagnosis of idiopathic pulmonary arterial hypertension was corrected by eukaryotic translation initiation factor 2 alpha kinase 4 (EIF2AK4) mutation screening. Biallelic mutations (c.1387delT (p. Arg463fs); c.989-990 delAA (p. Lys330fs)) were detected by next-generation sequencing for whole exome from blood sample. The presence of biallelic EIF2AK4 mutation was sufficient to confirm the diagnosis of PVOD.

**Interventions:**

The 2 patients had good response to PAH-targeted therapy (Ambrisentan 10 mg once a day and tadalafil 20 mg once a day) in the following 1 year.

**Outcomes:**

Because the patients had a good response to targeted drugs, the treatment of the 2 cases was unchanged. Over 1-year period, they still have a good response to PAH-targeted drugs. There was no sign of pulmonary edema.

**Lessons:**

All these results may indicate that PVOD is not so rare and typical findings of PVOD are lacking in some patients. EIF2AK4 mutation screening by next-generation sequencing maybe useful to differentiate PVOD from other PAH subtypes. PVOD is a heterogeneity population and different patients have different characteristics including response to PAH-targeted therapy. How to pick off this portion of patients timely is the core issue. Further study is necessary to answer this question.

## 1 Introduction

Pulmonary veno-occlusive disease (PVOD) is a kind of rare and fatal pulmonary arterial hypertension (PAH), which is difficult to diagnose and treat. It was first described by Dr. Julius Hora of Munich University in 1934 that PVOD is characterized by widespread occlusion of the pulmonary venules by fibrous tissue.^[1]^ The reported incidence rate of PVOD is 0.1 to 0.2 cases per million every year.^[2]^ Patients usually present with nonspecific respiratory and/or cardiac symptoms, including shortness of breath, dyspnea on exertion, fatigue, chest pain, dizziness, cough, and hemoptysis. PVOD is a clinicopathological entity with the diagnosis based on clinical manifestations and tissue confirmation. A combined picture of PAH along with concurrent pulmonary edema findings on diagnostic imaging is usually the initial hint toward PVOD diagnosis. The gold standard for diagnosis is lung biopsy, which usually shows pulmonary venules intimal fibrosis with diffuse smooth muscle narrowing, post-capillary proliferation, and interstitial/alveolar hemosiderophages.^[3]^ Biopsies are infrequent because patients often show intolerance or risk of bleeding.^[4]^ PVOD has a dismal prognosis and PAH-targeted drugs are not recommended. Lung transplantation is the most effective treatment.^[5]^ In this article, we present 2 cases of PVOD masquerading as idiopathic pulmonary arterial hypertension (IPAH) and responding well to PAH targeted drugs.

## 2 Case presentation

**Figure 1:**
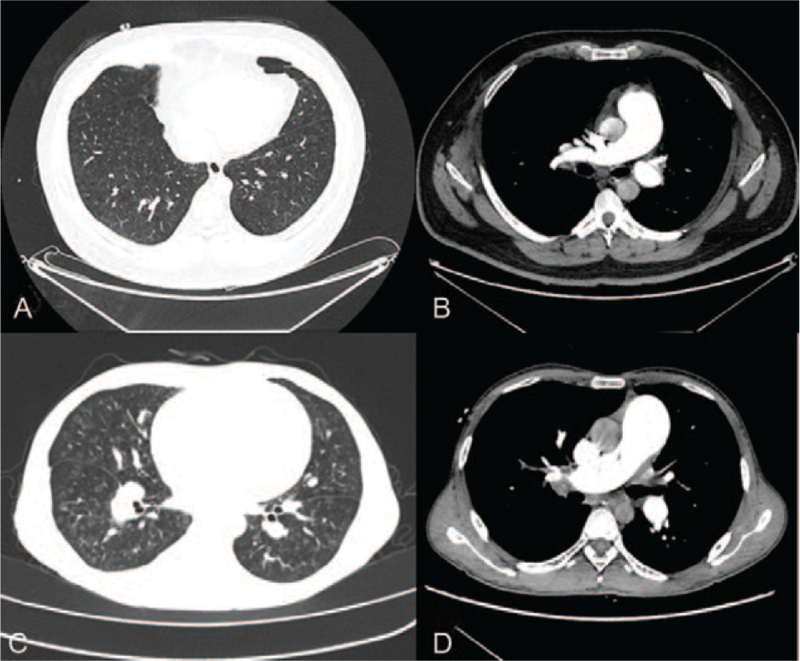
Representative chest CT obtained in the two biallelic EIF2AK4 mutation PVOD patients. First case (A and B): Chest CT revealed diffuse ground-glass opacities and obviously dilated main branch of pulmonary artery,second case (C and D): Chest CT revealed more severe ground glass opacity than in the first case and obviously dilated main branch of pulmonary artery.

**Figure 2:**
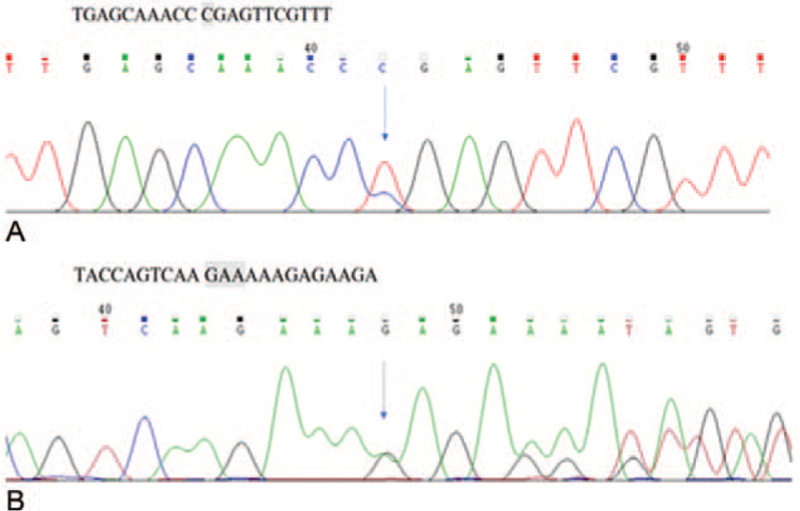
(A) First case EIF2AK4_ex9c.1387C > T (p. Arg463∗) Sanger sequencing validation map (positive, detected) (B) Second case EIF2AK4_ex8 c.989_990delAA (p. Lys330fs) Sanger sequencing validation map (positive, detected).

The first case, a 31-year-old male smoker, with no pertinent medical social, or family history presented was hospitalized for persistent cough and dyspnea for 6 months and progressive dyspnea for 1 week. He works as a bank clerk. The physical examination on admission showed that the body temperature was 36.7°C, the heart rate was 98/minute, the respiratory rate of 23/minute, and blood pressure was 132/89 mmHg. He had an accentuated second pulmonic heart sound. The 6-minutes walking distance was 360 m. The arterial blood gases test indicated that PaO_2_ was 48 mmHg and PaCO_2_ was 29.4mmHg in breathing room air. The results of the hematologic and biochemical tests were within the normal range. Laboratory workup was negative for HIV, antinuclear antibody, thyroid stimulating hormone, rheumatic factor, antineutrophil cytoplasmic antibody, and erythrocyte sedimentation rate et al. The concentration of *N*-terminal pro-brain natriuretic peptide was 4140 ng/mL. The transthoracic echocardiogram revealed a dilated right ventricle with a normal left ventricular function and an estimated right ventricular systolic peak pressure of 42 mmHg. Computed tomography (CT) pulmonary angiogram was negative for pulmonary embolism (PE), but showed ground-glass opacities and obviously dilated main branch of pulmonary artery (Fig. 1). No acute or chronic deep venous thrombosis was found in the deep vein color Doppler examination of lower extremity. Considering the possibility of PAH, right heart catheterization was done. The results showed severe PAH with mean pulmonary artery pressure at 76 mmHg, pulmonary artery wedge pressure at 14 mmHg, and cardiac output 2.4 L/min/m^2^. Therefore, the patient was initially diagnosed with IPAH and used PAH targeted drugs (Ambrisentan 10 mg once a day and tadalafil 20 mg once a day). After treatment, his symptoms of cough and dyspnea improved. Because the patient was diagnosed as IPAH and for better differential diagnosis, we tested the next-generation sequencing (NGS) of the whole exon group in the blood samples of the patients. A biallelic mutation was found in the coding sequence region in exon 9 of eukaryotic translation initiation factor 2 alpha kinase 4 (EIF2AK4), c.1387delT (p. Arg463fs)) (Fig. 2). According to the 2015 ESC/ERS Guidelines, the presence of biallelic EIF2AK4 mutation was sufficient to confirm the diagnosis of PVOD without a histological confirmation. Because the patient had a good response to targeted drugs, the treatment was unchanged. Over 1-year period, he still has a good response to PAH-targeted drugs. There was no sign of pulmonary edema. Lung CT scan was repeated and pulmonary edema was not detected (Table 1).

**Table 1:** Summarized of the treatment course of the first case.

Time	April 2020 pre-treatment post-treatment		May 2020	March 2021
SaO2 (%) (21% O_2_)	82	93	94	95
NT-pro BNP, ng/mL	4030	2350	414	210
6MWT, min	360	410	430	455
Functional class (NYHA)	III	II	II	II
PAH-targeted drugs	Ambrisentan + tadalafil	Ambrisentan + tadalafil	Ambrisentan + tadalafil	Ambrisentan + tadalafil

The second case was a 38-year-old male, who did not smoke and had no related medical history, social history, or family history. He was sent to the hospital because of persistent cough and dyspnea for 6 years and progressive dyspnea for 1 week. His job was a cooker. On admission, the physical examination showed a body temperature was 36.6°C, heart rate was 96 beats/minute, respiratory rate was 30 breaths/minute, and blood pressure was 101/78 mmHg. He had an accentuated second pulmonic heart sound. The 6-minutes walking distance was 350 m. The arterial blood gases test indicated that PaO_2_ was 47 mmHg and PaCO_2_ was 29 mmHg in breathing room air. The results of the hematologic and biochemical tests were within the normal range. Laboratory workup was negative for HIV, antinuclear antibody, thyroid stimulating hormone, rheumatic factor, antineutrophil cytoplasmic antibody, and erythrocyte sedimentation rate et al. The concentration of N-terminal pro-brain natriuretic peptide was 4480ng/mL. The transthoracic echocardiogram revealed a dilated right ventricle with a normal left ventricular function and an estimated right ventricular systolic peak pressure of 95 mmHg. The chest CT scan images showed ground-glass opacities (Fig. 1). Lower extremities duplex was negative for acute or chronic deep venous thrombosis. Considering the possibility of PH, right heart catheterization was done. The results showed severe pulmonary arterial hypertension with mean pulmonary artery pressure at 68 mmHg, pulmonary artery wedge pressure at 11mmHg, and cardiac output 2.8 L/min/m^2^. The patient was diagnosesd as IPAH initially and PAH-targeted drugs (Ambrisentan 10 mg once a day and tadalafil 20 mg once a day) were used also. After treatment, dyspnea improved. NGS for whole exome was also tested in blood samples of the patient. A biallelic mutation was found in the coding sequence region in exon 9 of EIF2AK4 (c.989-990 delAA [p. Lys330fs]) (Fig. 2). So the diagnosis of PVOD was confirmed. Because he had a good response to the drugs, the treatment was unchanged. Over a period of >20 months, he has a good response to PAH-targeted drugs. No signs of pulmonary edema were found. Lung CT scan was repeated and pulmonary edema was not detected (Table 2).

**Table 2:** Summarized of the treatment course of the second case.

Time	September 2019 pre-treatment	Post-treatment	March 2020	September 2020	March 2021
SaO2 (%) (21% O_2_)	84	88	93	94	93
NT-pro BNP, ng/mL	3630	1080	760	510	563
6MWT, min	280	320	362	447	430
Functional class (NYHA)	IIII	III	III	II	II
PAH-targeted drugs	Ambrisentan + tadalafil	Ambrisentan + tadalafil	Ambrisentan + tadalafil	Ambrisentan + tadalafil	Ambrisentan + tadalafil

Standard care is performed for these 2 patients, so ethical approval is not applicable in this study. Written informed consent was obtained from the patients.

## 3 Discussion

PVOD represents a rare form of PAH characterized by preferential involvement of the pulmonary venous system.^[6,7]^ The pathological hallmark is obliteration of small pulmonary veins by fibrous intimal thickening and patchy capillary proliferation.^[8]^ Similar to other types of obstructive pulmonary vascular diseases, PVOD results in a progressive increase in pulmonary vascular resistance, which eventually leads to right heart failure and death.^[9]^ More than 80 years ago, German doctor J. Hora first described the clinical and pathological characteristics of PVOD in detail.^[1]^ However, it was not until 1966 that HEATH et al invented the term PVOD,^[10]^ and they realized that PVOD was different from the so-called “primary pulmonary hypertension”. Recently, we have achieved an important milestone in the study of PVOD, and found that the biallelic mutation of EIF2AK4 gene leads to heritable PVOD.^[11]^ Despite significant strides in our knowledge of the genetic, cellular and molecular basis of PVOD over the past decade, it remains classically an orphan lung disease.

The etiology of PVOD is still unclear, but it is related to genetic factors, anticancer drugs such as mitomycin and bleomycin, bone marrow transplantation, radiotherapy, smoking, connective tissue diseases such as systemic lupus erythematosus and rheumatoid arthritis, and there are also reports of PVOD caused by chronic hepatitis.^[12]^ French pulmonary hypertension network reported 13 PVOD family pedigrees with hereditary diseases, and proved that EIF2AK4 had double allele mutation.^[11]^ French PH network pointed out that when they provided genetic counseling and EIF2AK4 mutation screening to all patients with or without family history, these mutations were also found in 9% sporadic PVOD cases.^[13]^ The present 2 patients had no family history of PVOD. The screening tests for connective tissue diseases s were all negative. Although smoking is considered to be a risk factor, there is no conclusive evidence.

The diagnosis of PVOD is confirmed by histological examination of either postmortem or explanted lung tissue in previous. Elevated pulmonary venous and arterial pressures may result in a risk of life-threatening bleeding. Lung biopsy is not tolerated and is not recommended. Data from whole genome sequencing from a recent investigation demonstrated that mutations in EIF2AK4 were present in all familial PVOD and in 25% of the sporadic PVOD cases. The results support the conclusion that EIF2AK4 is a major causal gene for PVOD.^[11]^ The 2015 ESC/ERS guidelines recommend that patients with sporadic or familial PVOD should be tested for EIF2AK4 mutation. The presence of a biallelic EIF2AK4 mutation is sufficient to confirm the diagnosis of PVOD without the need for dangerous lung biopsy for histological confirmation.^[1]^ Therefore for a PAH patient with clinical suspicion of PVOD, biallelic EIF2AK4 variant testing is recommended, regardless of familial history.^[14]^ The discovery of biallelic EIF2AK4 mutations raised the possibility of rapid molecular diagnosis for PVOD.

PVOD shares several clinical and hemodynamic similarities with IPAH, thus often leading to a misdiagnosis as IPAH.^[15,16]^ It has some characteristics on CT scan. Typical findings from high-resolution CT of the chest are the presence of centrilobular ground-glass opacities, subpleural thickened septal lines, and mediastinal lymphadenopathy.^[17]^ The chest CT scan images of the two patients showed only ground-glass opacities and dilated main branch of pulmonary artery. Absent of these manifestations lead to the diagnosis of IPAH at first, in the present two patients. Fortunately, NGS for whole exome was routinely tested for PAH patients in our center. These results may also indicate that PVOD is not so rare and typical finding of PVOD is lack in some patients.

Presently approved PAH drugs target one of the three major pathways involved in PAH pathogenesis: the prostacyclin, endothelin-1, and NO pathways.^[18]^ All PAH drugs have vasodilatory properties and also variable antiproliferative effects on the pulmonary vasculature.^[19]^ However, data on the use of PAH therapy in PVOD are weak and conflicting. Case reports/series and anecdotal experience suggests that few patients with PVOD can achieve hemodynamic and functional improvement or at least stabilize the course of disease through PAH targeted therapy.^[20]^ However, life-threatening pulmonary edema is the main complication of pulmonary vasodilatory therapy in PVOD, and can occur with any class of PAH drugs. Prevalence of pulmonary edema may be as high as 50% following initiation of PAH-targeted drugs.^[21]^ PAH therapies should only be conducted in experienced pulmonary vascular centers with experience in the management of this condition. The most open experience of PAH treatment in PVOD is with the use of i.v. epoprostenol in 12 patients with severe PVOD as a bridge to transplantation.^[22]^ Epoprostenol was given at low dose ranges (median peak dose of 13 ng/kg/min) with slow up-titration of the dose. High-dose loop diuretics are required to minimize the risk of pulmonary edema. Moderate clinical and hemodynamic improvement may be observed after 3 to 4 months. Mild clinical improvement or stabilization of disease has also been reported with other PAH drugs such as bosentan,^[20]^ sildenafil,^[23]^ and iloprost. Increasingly, upfront combination therapy is also adopted for PAH therapy in PVOD patients, particularly if high-risk features are present.^[24]^ Careful monitoring must be conducted when PAH therapies are used in cases of PVOD.

Luo et al also reported that none of the patients with clinically diagnosed PAH and biallelic EIF2AK4 mutations developed pulmonary edema in response to pulmonary artery vasodilator therapies.^[25]^ In the typical PVOD patients, the incidence of pulmonary edema with intravenous prostanoids has been reported as high as 44% after a median treatment duration of just 9 days. It is speculated that the degree and severity of pulmonary vein involvement in these patients may be the basis of different responses to prostaglandins.^[26]^ Nossent et al reported that in addition to typical veno-occlusive lesions, substantial pulmonary arterial lesions, and important microvascular remodeling were also found in 24 cases of PVOD.^[27]^ These PVOD patients had pathological characteristics similar to PAH. These may contribute to the good response to vasodilators in our patients.

In the study, we reported 2 cases of PVOD which was misdiagnosed as IPAH initially due to the lack of typical findings of PVOD. Fortunately, the diagnosis was corrected by EIF2AK4 mutation screening and biallelic mutations were detected. The 2 patients had good response to PAH-targeted therapy. All these results may indicate that PVOD is not so rare and typical findings of PVOD are lacking in some patients. EIF2AK4 mutation screening by NGS may be useful to differentiate PVOD from other PAH subtypes. PVOD is a heterogeneity population and different patients have different characteristics including response to PAH-targeted therapy. How to pick off this portion of patients timely is the core issue. Further study is necessary to answer this question.

## Author contributions

**Conceptualization:** Yao Wang.

**Data curation:** li zhang.

**Funding acquisition:** Ruifeng Zhang.
